# Acute kidney injury prediction in cardiac surgery patients by a urinary peptide pattern: a case-control validation study

**DOI:** 10.1186/s13054-016-1344-z

**Published:** 2016-05-26

**Authors:** Jochen Metzger, William Mullen, Holger Husi, Angelique Stalmach, Stefan Herget-Rosenthal, Heiner V. Groesdonk, Harald Mischak, Matthias Klingele

**Affiliations:** Mosaiques Diagnostics GmbH, Hannover, Germany; Institute of Cardiovascular and Medical Sciences, Glasgow, UK; Department of Medicine and Nephrology, Rotes Kreuz Krankenhaus, Bremen, Germany; Department of Anaesthesiology, Intensive Care and Pain Medicine, Saarland University Medical Centre, Homburg-Saar, Germany; Department of Internal Medicine, Nephrology and Hypertension, Saarland University Medical Centre, Homburg-Saar, Germany; Department of Internal Medicine, Hochtaunus-Kliniken, Usingen, Germany

**Keywords:** Acute kidney injury, Cardiac surgery, Urinary peptide pattern, Prediction, Test validation

## Abstract

**Background:**

Acute kidney injury (AKI) is a prominent problem in hospitalized patients and associated with increased morbidity and mortality. Clinical medicine is currently hampered by the lack of accurate and early biomarkers for diagnosis of AKI and the evaluation of the severity of the disease.

In 2010, we established a multivariate peptide marker pattern consisting of 20 naturally occurring urinary peptides to screen patients for early signs of renal failure. The current study now aims to evaluate if, in a different study population and potentially various AKI causes, AKI can be detected early and accurately by proteome analysis.

**Methods:**

Urine samples from 60 patients who developed AKI after cardiac surgery were analyzed by capillary electrophoresis-mass spectrometry (CE-MS). The obtained peptide profiles were screened by the AKI peptide marker panel for early signs of AKI. Accuracy of the proteomic model in this patient collective was compared to that based on urinary neutrophil gelatinase-associated lipocalin (NGAL) and kidney injury molecule-1 (KIM-1) ELISA levels. Sixty patients who did not develop AKI served as negative controls.

**Results:**

From the 120 patients, 110 were successfully analyzed by CE-MS (59 with AKI, 51 controls). Application of the AKI panel demonstrated an AUC in receiver operating characteristics (ROC) analysis of 0.81 (95 % confidence interval: 0.72–0.88). Compared to the proteomic model, ROC analysis revealed poorer classification accuracy of NGAL and KIM-1 with the respective AUC values being outside the statistical significant range (0.63 for NGAL and 0.57 for KIM-1).

**Conclusions:**

This study gives further proof for the general applicability of our proteomic multimarker model for early and accurate prediction of AKI irrespective of its underlying disease cause.

**Electronic supplementary material:**

The online version of this article (doi:10.1186/s13054-016-1344-z) contains supplementary material, which is available to authorized users.

## Background

Acute kidney injury (AKI) is a common complication among hospitalized patients occurring in 5 % of all hospital and 35 % of intensive care unit (ICU) admissions [1, 2]. Moreover, AKI considerably increases morbidity, mortality and health care expenditures, independently of other comorbidities [1]. Patients undergoing cardiac surgery have a highly increased risk to develop postoperative AKI compared to other hospitalized patients. This is attributed to procedure-related factors such as the use of extracorporeal circulation or aortal clamping time, potentially leading to exacerbated in situ hypoxic conditions, and resulting in up to 30 % incidences of postoperative AKI [2, 3]. Moreover, occurrence of AKI plays an important role for the postoperative outcome, being associated with high mortality [4]. Therefore, detection of AKI is of major interest especially for patients undergoing cardiac surgery and other extensive surgery regimes.

The recent Acute Kidney Injury Network (AKIN) definition and classification of AKI incorporates serum creatinine and urine output as markers to diagnose and stage AKI [5]. However, these markers neither permit early detection of AKI nor indicate the underlying pathophysiological mechanisms.

Although no effective therapy of AKI is currently available, its earliest possible diagnosis seems to be advantageous since postoperative AKI is associated with mortality after cardiac surgery [3].

Proteomics is the part of omics technologies that most specifically displays an individual’s physiological state. The proteome is encoded by the genome in alteration to external signals and modulants, and is further diversified by posttranslational modifications and proteolytic processing. Compared to blood, the urine proteome is less complex and to a lesser extent subject to further dynamic alterations due to a low proteolytic activity in situ making it more stable ex vivo [6–8]. The proteomic content of urine arises from the secretion and shedding of proteins from the kidney endothelium, release of kidney epithelial exosomes inside the kidney, and also glomerular filtration and endosomal-exosomal passage of blood-circulating proteins. Capillary electrophoresis-mass spectrometry (CE-MS) has emerged in recent years as a hybrid technology using capillary electrophoresis (CE) instead of liquid chromatography for protein separation before mass spectrometry (MS) [9]. Without the need of a sieving matrix, CE-MS enables sensitive (1 fmol) and fast (approximately 1 h) analysis of the low molecular weight proteome in the range of 0.8–20 kDa as demonstrated in a large number of studies as recently reviewed by Pejchinovski et al. [10].

In the last years, several urinary compounds (e.g., neutrophil gelatinase-associated lipocalin [NGAL]) were shown to be able to detect AKI earlier compared to increase of serum creatinine according to the currently used definition and classification of AKI [1, 4]. However, these single markers are related to a specific pathophysiological process of AKI, e.g., NGAL to ischemic or toxic tubular damage [5, 11, 12]. Since reasons for acute renal failure in clinical practice are very heterogeneous, this may explain conflicting results in the literature concerning those single-marker tests for early detection of AKI. However, those biomarkers were shown to have the capacity to detect early AKI and also the predictive capacity for extrarenal postoperative complications, e.g., nonocclusive mesenterial ischemia (NOMI) or death [13]. We therefore compared the predictive capacity of proteome analysis for AKI with NGAL and kidney injury molecule-1 (KIM-1), and we evaluated whether extrarenal complications could have influenced the detection of AKI. Therefore, a method of detecting AKI independent of the underlying pathogenesis or other extrarenal occurring complications seems promising. In our previously published pilot study [14], we used CE-MS to establish a urinary peptide test predictive for AKI. Twenty polypeptides from the urinary proteome were selected as a panel of markers to generate a classifier. The marker pattern was able to detect early AKI and was superior in prediction of AKI compared to serum cystatin C, urinary kidney injury molecule-1 (KIM-1), interleukin-18 (IL-18) or NGAL.

The current study has the following objectives: first, the validation of the urine peptide marker panel in a different clinical setting to prove its prognostic accuracy for AKI independently of the underlying pathogenesis. Second, the comparison of the urine peptide marker panel’s classification performance in this group of post-cardiac surgery patients to that based on urinary NGAL and KIM-1 levels. Third, the evaluation of the urine peptide marker panels predictive capacity with respect to the severity of AKI, and finally association of proteomic classification to the AKI-associated complications, NOMI, and death.

## Methods

### Patients and samples

For this study, samples of 120 patients were retrospectively selected from a large prospective cohort study population of 865 patients undergoing cardiac surgery aiming to identify prognostic markers for postoperative complications. This prospective study and the included population is described in detail in Speer et al. [15] and Klingele et al. [16]. Within this study, AKI was defined according to the Acute Kidney Injury Network (AKIN) criteria [5]. Written informed consent had been obtained from all patients. Ethical approval was obtained from the local ethics committee (Landesärztekammer des Saarlandes; Ref. ID: 199/09) and the study conformed to the standards set by the Declaration of Helsinki.

Since the study took place 2 years after discharge of patients, clinical data and outcome were known, especially whether or not AKI occurred, and also included knowledge about the severity of AKI and AKIN class. Moreover, since this validation study aimed to investigate whether our previously published proteome test can accurately predict AKI in a clinical setting, the sole selection criterion was to obtain a cohort of 60 patients with AKI being balanced in the distribution of the different AKIN classes. For this reason, we randomly selected 25 patients out of all patients with AKIN 1. In the same way 15 patients with AKIN 2 and 20 patients with AKIN 3 were chosen. From the patient pool without AKI we randomly picked 60 patients as a control group.

For preoperative analysis, blood was drawn from the central venous catheter and urine samples were collected from the urinary catheter both directly before the intervention. The surgical procedures were generally carried out during the day. The next morning at 6:00 am, between 12 to 18 hours after surgery, postoperative samplings of blood and urine were performed. Urine samples were transported on ice, aliquoted in polypropylene tubes (Sarstedt AG, Nümbrecht, Germany) and stored at –80 °C for subsequent analysis.

Proteome analysis was carried out by Mosaiques Diagnostics GmbH in Hannover as previously published [14]. Analysis took place 2 years after cardiac surgery. Samples alone without clinical information related to the patients were sent, and the proteome analyses were therefore performed in a blinded manner. Subsequent to communicating the results of the proteome analyses to the physicians at the Saarland University Hospital, samples were all unblinded and all clinical data necessary for further evaluation (e.g., AKI or no AKI) were provided.

### Sample preparation

The urine samples were prepared as previously described by removing large proteins (>20 kDa), urea, electrolytes and salts, and by enriching polypeptides [17]. Briefly, 700 μL of urine were defrosted with the addition of 0.1 % PMSF saturated in ethanol and diluted with 700 μL of a solution containing 2 M urea, 0.1 M NaCl, 10 mM NH_4_OH and 0.02 % SDS. The mixture was then filtered through a 20 kDa MW cut-off ultracentrifugation filter device (Sartorius Stedim UK Ltd, Stonehouse, United Kingdom) at 3000 × g for 1 hour at 4 °C. A volume of 1.1 mL of the resulting filtrate was loaded onto a pre-equilibrated PD-10 desalting column (GE Healthcare, Stockholm, Sweden) and eluted using 0.01 % aqueous NH_4_OH. The eluate was subsequently freeze-dried and stored at 4 °C prior to being resuspended in HPLC-grade water to a final protein concentration of 2 mg/mL for CE-MS analysis.

### CE-MS analysis

CE-MS analysis was performed as described by Metzger et al. [14] using a P/ACE MDQ capillary electrophoresis system (Beckman Coulter, Fullerton, CA, USA) on line coupled to a MicroTOF MS (Bruker Daltonic, Bremen, Germany). Samples were injected hydrodynamically at 2.0 psi for 99 sec (ca. 250 nL) and separation of peptides was achieved by reverse polarity at 25 kV for the first 30 min, and with increasing pressure (up to 0.5 psi) for another 34 min. The cartridge temperature was maintained at 25 °C. Running buffer contained 79:20:1 (v/v) MilliQ water, acetonitrile, and formic acid. Sheath liquid consisted of 30 % 2-propanol and 0.4 % formic acid in MilliQ water. The ESI sprayer (Agilent Technologies, Palo Alto, CA, USA) was grounded, and the ion spray interface potential was set between -4 and -4.5 kV. Spectra were accumulated every 3 seconds over a range of mass-to-charge ratios from 50 to 3000. Details on accuracy, precision, selectivity, sensitivity, reproducibility, and stability of the CE-MS method have been previously reported [8, 17–19].

### Proteomic data processing and sample classification

Mass spectral ion peaks with a charge >1 and a signal-to-noise ratio >4 detectable in at least three consecutive spectra and assignable to the same peptide entity were deconvoluted into single masses using the MosaiquesVisu software [20, 21]. CE migration time and ion signal intensity were normalized using internal peptides with high abundance and low mass deviation as calibrants [22]. Sample-specific peptide lists were deposited in a SQL database for subsequent analysis.

Test results of the samples were classified into the categories ‘AKI’ and ‘non-AKI’ by the urinary AKI predictive peptide marker panel, the classification algorithm and the cut-off point at 0.02. The later was established in our recent pilot study as the classification score with the highest average in sensitivity and specificity on a representative ICU training set [14].

### Immunoassays and laboratory tests

NGAL and KIM-1 were measured in urine samples using human-specific ELISA assays (Enzo Life Sciences, Exeter, UK; kits #BPD-KIT-036 and #ADI-900-226-0001 respectively) and were carried out according to the manufacturer’s instructions. Modifications of recommended assay conditions included higher dilutions for both kits to cover a higher than expected quantity range in neat urine.

### Statistical methods

Estimates of sensitivity and specificity for the AKI predictive peptide marker model were calculated by tabulating the number of correctly classified samples. Confidence intervals (95 % CI) for sensitivity and specificity were based on exact binomial calculations and were carried out in MedCalc 12.7.7.0 (MedCalc Software, Mariakerke, Belgium) as were the receiver operating characteristic (ROC) plots. The area under the ROC curve (AUC) was evaluated, as it provides a single measure of overall accuracy independent upon a particular threshold [23].

## Results

The selected 60 AKI case and 60 non-AKI controls were analyzed by CE-MS to obtain their proteomic profiles in the mass range of 0.8–20 kDa. One AKI case and nine non-AKI control samples failed to fulfill the quality control criteria for the CE-MS profiles due to the presence of considerable amounts of polymers in the catheter urine and were therefore excluded from further analysis.

An overview of the patient’s demographic and clinical data in the two groups relevant to this study is presented in Table 1. Since the occurrence of AKI was the sole criterion for selection, AKI patients and those without AKI are significantly different with respect to age, preoperative renal function or the occurrence of severe postoperative complications such as NOMI or death. A total of 25 % of patients with AKI required renal replacement therapy.

**Table 1:** Clinical and demographic data of AKI cases and non-AKI controls of post-cardiac surgery patients

Parameter	AKI	Non-AKI	*p*
Patients/samples (n/n)	59/59	51/51	
Age (years)^†^	65 (45–77)	60 (24–77)	0.03
Gender (female/male)	13/46	16/35	0.29
EuroSCORE II^**^ (points)^†^	6.82 (0.86–28.34)	5.99 (0.67–21.93)	0.89
Body mass index^†^	30.7 (19.9–47.3)	30.8 (17.3–41.4)	0.86
S-creatinine at baseline (mg/dL)^†^	1.1 (0.6–1.9)	0.9 (0.6–1.3)	<0.001
Estimated glomerular filtration rate at baseline (mL/h/1.73 m^2^)^‡,†^	73 (37–110)	92 (56–132)	<0.001
Diabetes (%)	29	18	0.19
OP time (min)^†^	214 (105–390)	211 (109–651)	0.6
Time of cardiopulmonary bypass (min)^†^	109 (48–243)	103 (34–177)	0.81
Clamping time (min)^†^	66 (17–184)	67 (26–125)	0.77
Endpoints:			
NOMI (%)^ǁ^	29	2	<0.001
Death (%)^ǁ^	17	0	0.002
AKI staging (n):			
0	0	51	
1	25	0	
2	14	0	
3	20 (15 w/ hemodialysis)	0	

*AKI* acute kidney injury, *NOMI* nonocclusive mesenteric ischemia,

^**^Thoracic Surgeons Risk Score to predict mortality after thoracic surgery, for calculation see: http://www.euroscore.org/

^‡^According to the Chronic Kidney Disease Epidemiology Collaboration (CKD-EPI)

^†^Given as mean (range)

^ǁ^7 % of AKI patients with composite endpoint

All CE-MS profiles that passed the quality control were classified using the AKI predictive peptide marker model as described previously [14]. CE-MS characteristics and sequence information of the 20 peptides included in this peptide marker model are presented in Additional file 1: Table S1.

**Fig. 1:**
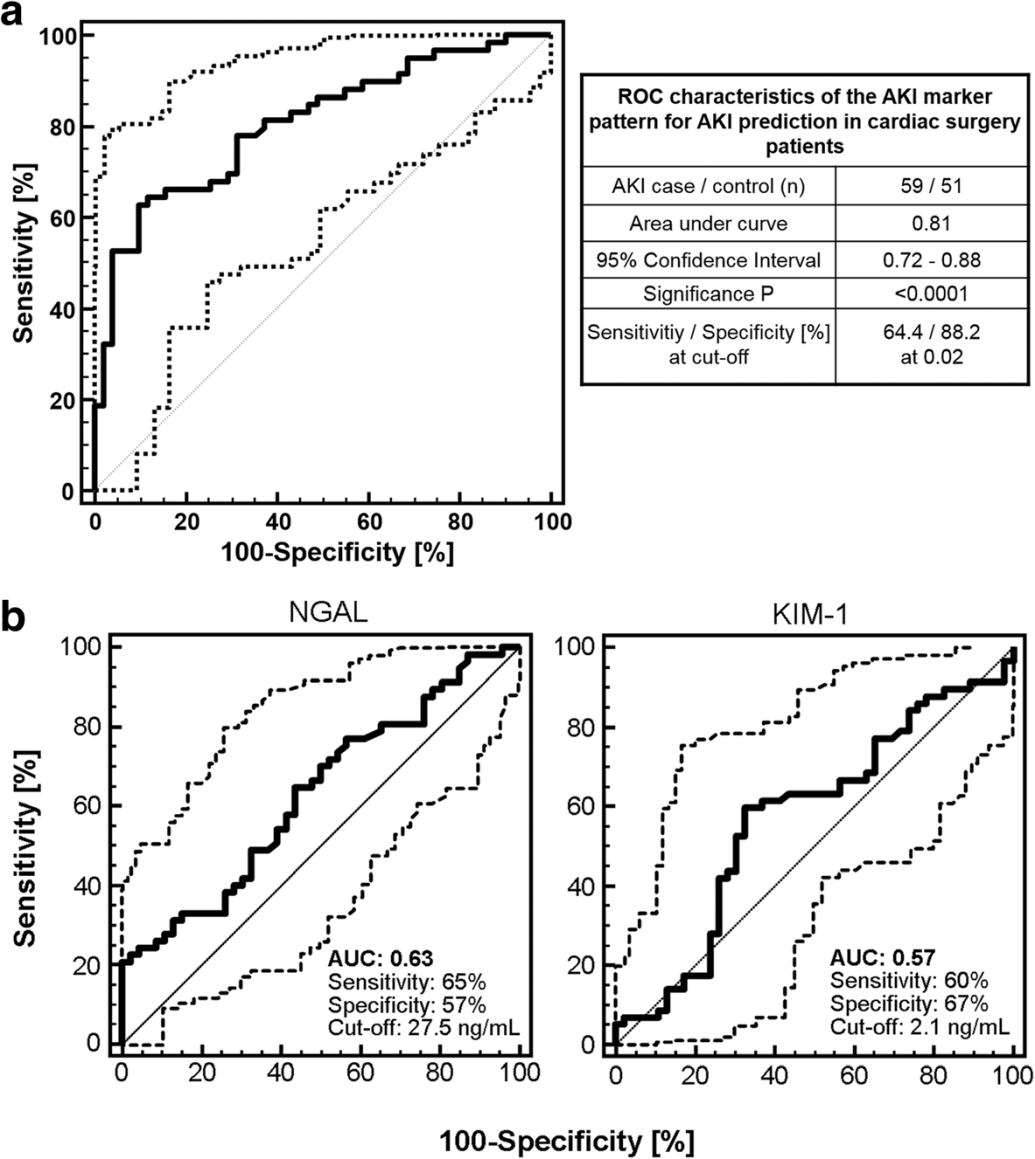
ROC characteristics for prediction of AKI in post-cardiac surgery patients (**a**) by the AKI peptide marker panel and (**b**) by the single markers NGAL and KIM-1. Whereas for the AKI peptide marker panel the sensitivity and specificity was determined at the previously established cut-off, these values were determined for NGAL and KIM-1 at the optimal cut-off according to the Youden index. *AKI* acute kidney injury, *AUC* area under the ROC curve, *KIM-1* kidney injury molecule-1, *NGAL* neutrophil gelatinase-associated lipocalin, *ROC* receiver operating characteristic

Classification performance of the AKI predictive model for the study cohort of cardiac patients was evaluated by ROC analysis [23]. As shown in Fig. 1a, the AKI peptide marker model enabled prediction of AKI with an AUC value of 0.81 and a range within the 95 % confidence interval (CI) from 0.72 to 0.88, which is highly significant (*p* < 0.0001). At the predetermined cut-off of 0.02, the sensitivity and specificity of the AKI peptide marker model on this post-cardiac surgery patient cohort was 64.4 and 88.2 %, respectively. In comparison to the AKI peptide marker model, the proposed single AKI markers NGAL and KIM-1 as determined by ELISA only showed low classification accuracy on the patient set with AUC values of 0.63 (95 % CI: 0.53–0.72; *p* = 0.02) for NGAL and 0.57 (95 % CI: 0.47–0.67; *p* = 0.22) for KIM-1 (Fig. 1b). The AUC of the ROC curve of the AKI peptide marker model was significantly superior to both NGAL (*p* = 0.0032) and KIM-1 (*p* = 0.0034).

**Fig. 2:**
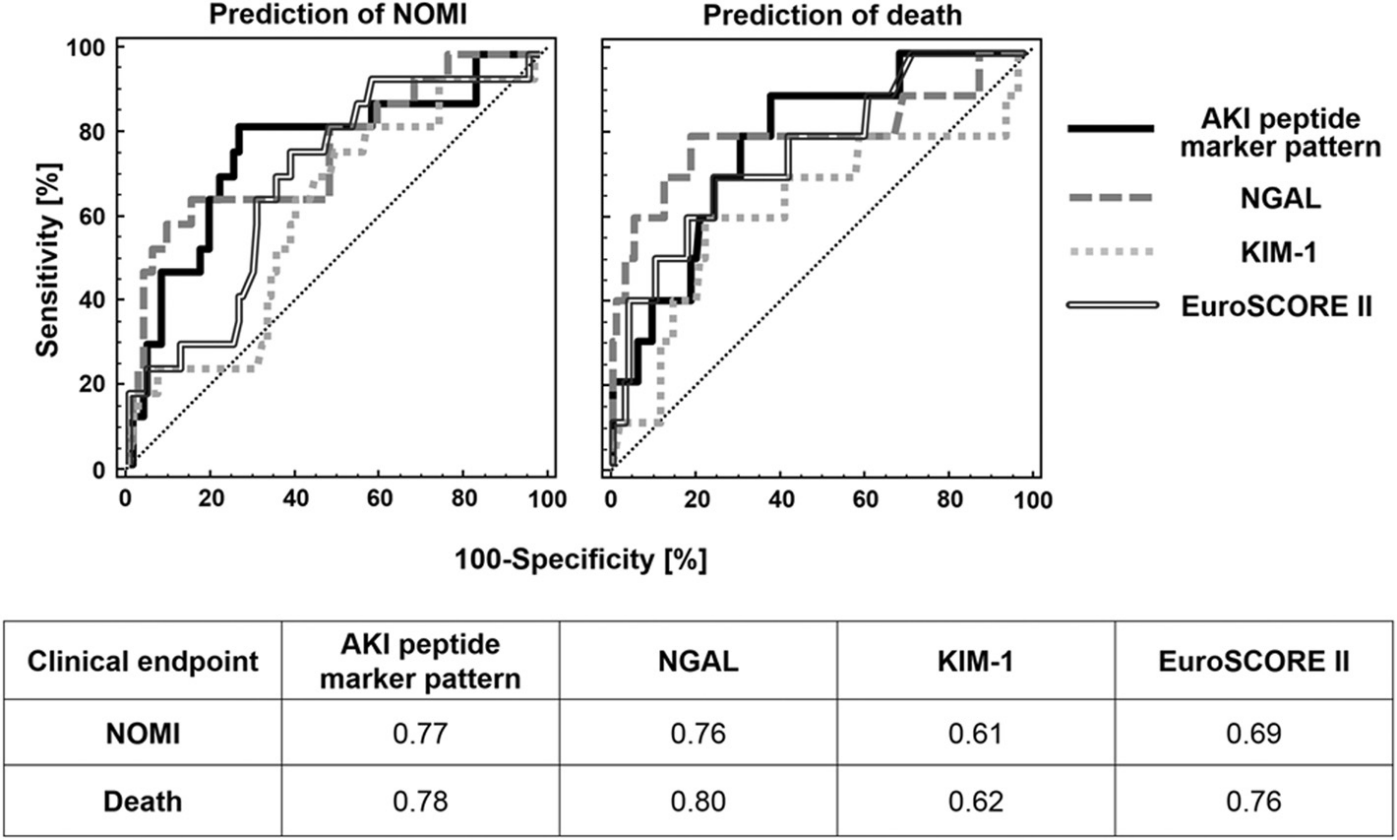
Classification accuracy of the AKI peptide marker pattern and the other AKI predictive markers NGAL, KIM-1 and EuroSCORE II for the AKI-associated endpoints nonocclusive mesenteric ischemia (NOMI) and death in comparative ROC analysis. Shown is a receiver operating curve (ROC) comparison of the different prognostic markers for the endpoints NOMI (*upper left panel*) and death (*upper right panel*) and a table summarizing the corresponding ROC-derived area under the curve (AUC) values of the different prognostic markers for these two AKI-associated endpoints. *AKI* acute kidney injury, *KIM-1* kidney injury molecule-1, *NGAL* neutrophil gelatinase-associated lipocalin

We tested if urine proteomics would be predictive for other postoperative complications such as NOMI or death (Fig. 2). NGAL and our urine peptide marker model showed comparable predictive capacity for NOMI and death. Interestingly, both NGAL and our urine peptide marker model were at least as predictive for postoperative mortality as the 18 factors comprising EuroSCORE II being developed specifically for prediction of the 30-day mortality rate after cardiac surgery [24].

**Fig. 3:**
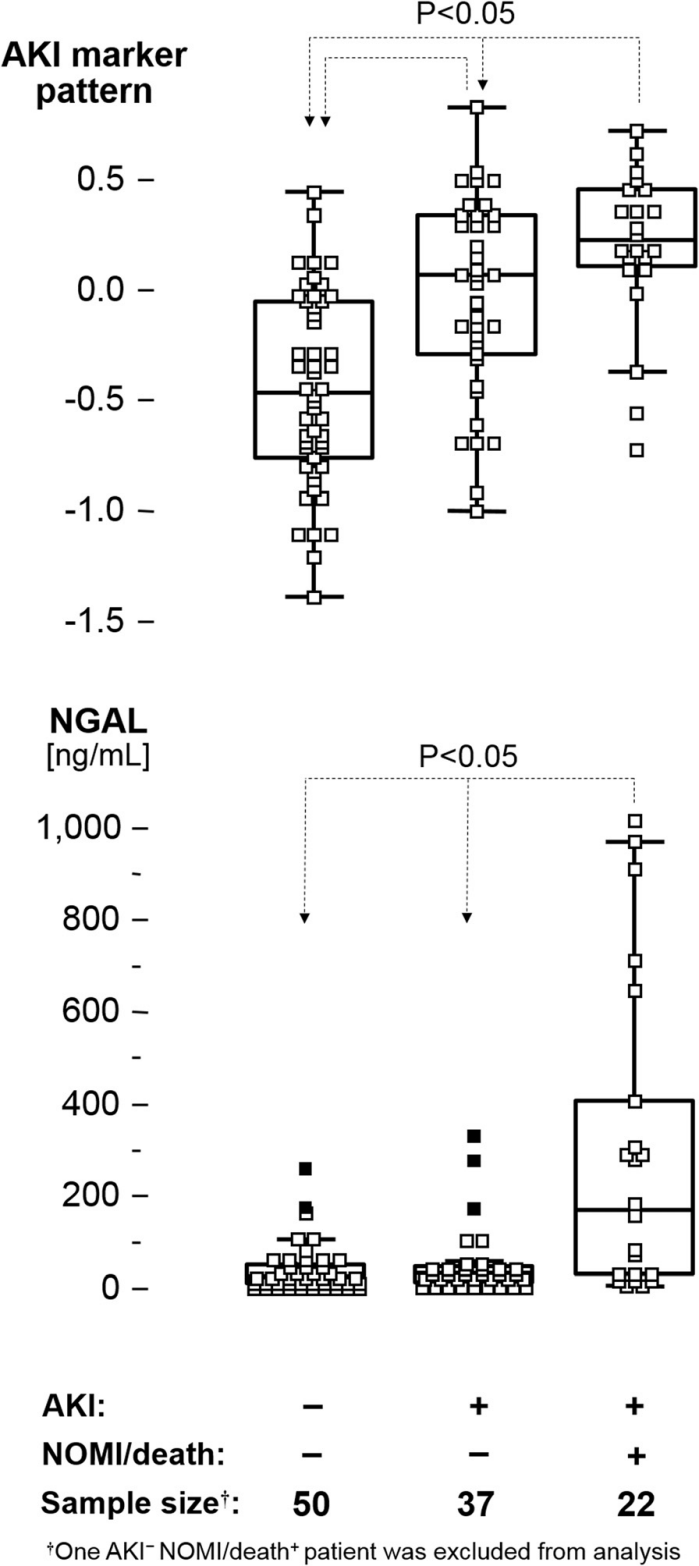
Distribution of classification scores of the AKI marker pattern (*upper panel*) and NGAL levels in ng/mL (*lower panel*) in AKI and non-AKI patient subgroups showing either progression or lack of nonocclusive mesenteric ischemia (NOMI) or death. A post hoc test was performed for average rank differences between the different subgroups (each with *p* < 0.05) after a significant result in the global Kruskal-Wallis test (*p* < 0.0001). *AKI* acute kidney injury, *NGAL* neutrophil gelatinase-associated lipocalin

Patients with postoperative AKI had significantly more additional serious complications. Therefore, we subdivided our cohort in three groups: no AKI and AKI, both without any other complication, and patients with AKI and with NOMI and/or death (Fig. 3). NGAL differentiated well between patients without AKI and those with AKI and NOMI or death. However, patients suffering only from AKI showed similar NGAL levels than those without AKI. In contrast, our urine peptide marker model differentiated patients without AKI from both AKI patients with as well as without NOMI and/or death.

**Fig. 4:**
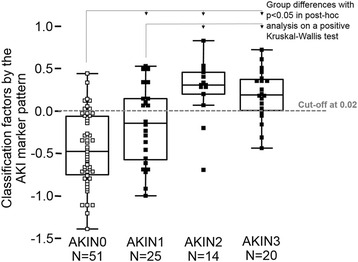
Box-and-whisker representation of classification factors for the different AKI stages by the AKI peptide marker pattern. *AKI* acute kidney injury

In order to evaluate whether the classification scores were associated with the severity of AKI according the AKIN criteria [5], a nonparametric rank sum test with the AKI stages as ordinal data was applied to the classification of patient samples by the proteomic test. As shown in the box-and-whisker plot of Fig. 4, significant rank sum differences were observed for patients without AKI (stage 0) in comparison to the other AKI stages and for stage 1 in comparison to stage 2 and 3. However, our results show overlaps especially for AKIN 2 and 3. Therefore, the classification scores of our AKI peptide marker model allow an early and accurate detection of AKI. Nevertheless, a reliable prediction of severity of AKI was not possible in all patients.

## Discussion

This study confirms the prognostic potential of our recently described urinary biomarker panel for the early detection of AKI [14]. In this respect, the performance of the proteomic classifier was superior to the single ELISA markers NGAL and KIM-1 in our validation patient cohort consisting of patients after cardiac surgery. This is in line with several studies showing conflicting results for the diagnostic performance of these markers. On the one hand, these single molecules were shown as potential biomarkers for AKI [25, 26]. On the other hand, in several other studies they failed to demonstrate any true benefit [14, 27–30]. A possible explanation for these opposing results would be that AKI represents a final result of many and divergent pathophysiological processes and causes, but that its diagnosis is uniformly based on increased serum creatinine and/or reduced urine output. This may explain why NGAL recognizes well and early AKI in cases of mainly tubular damage [11, 12] but less well in case of other pathophysiological mechanisms of AKI. Thus, detection of AKI by a panel of different peptides as proposed in our proteomic test seems to be more reliable covering numerous underlying pathological mechanisms.

Our results clearly demonstrate, that proteome analysis of the urine within 12 to 18 hours postoperatively allows accurate detection of postoperative AKI. Since AKI in this patient collective was induced by the surgical procedure, the diagnostic performance of the proteomic model was determined for AKI patients with a broad range of the time intervals between sampling and clinical diagnosis of AKI. AKI is defined by AKIN criteria [5]. Based on this definition, AKI can be clinically diagnosed in case of oliguria and anuria. However, when urine output remains unchanged, AKI only can be diagnosed and classified by the rate of increased serum creatinine levels within 48 hours [5]. This definition is in contrast to the currently pursued therapeutic principle of an “early goal-directed therapy”. Proteome analysis accelerates the detection of AKI and would support this therapeutic request. Our proteomic marker will meet these requirements of a rapid and reliable diagnosis of severe AKI regardless of the underlying cause. Although our urine peptide marker panel enabled accurate prediction of AKI in patients after cardiac surgery, reliable prediction of severity of AKI as expressed by the AKIN criteria was not possible in all of the patients, especially for the discrimination between AKIN 2 and 3. This could partially be explained by the small number of patients. Beyond that, the primary intention for investigating the urine proteome was the detection of AKI in general. The 20-peptide marker panel was developed for this purpose [14]. Therefore, in this study we tested for the first time if our proteomic test would also allow prediction of AKI severity. Since the results suggest an association of our classification scores with AKI severity, further development of our proteomic classifier could be helpful to improve the power of discrimination of AKIN classes. This hypothesis will be tested in further studies with sufficiently large study populations.

The complex causes and pathophysiological processes resulting in clinical AKI are not fully understood so far. Interestingly, many serious complications occur only in patients who also developed AKI. This leads to the question whether AKI is a cause for further complications or whether there may be a common pathogenetic mechanism, resulting in AKI and other complications. Since NOMI and death were primary endpoints in the prospective cohort study [15, 16] we used these clinical data for the former raised question.

Patients with postoperative AKI had significantly more other serious complications and our urine proteomic test and also NGAL were predictive for these postoperative complications, Therefore the predictive capacity for death or NOMI could be partially explained by this coincidence. However, when we assessed separately on complications and combination of AKI, NOMI, and/or death we could demonstrate that our test could well differentiate between patients developing AKI and those who did not. In contrast, NGAL was only able to do so in patients with AKI and NOMI. We hypothesized that similar pathophysiological mechanisms may trigger AKI and NOMI resulting in a high coincidence. These pathophysiological mechanisms result in tubular damage, explaining why NGAL detects these cases accurately. Thus, the underlying pathophysiological causes of AKI are different in patients who did not develop NOMI. This also would explain why AKI in these patients was not detected by NGAL, but with our urine peptide marker panel.

In view of the complexity of AKI, a multifactorial diagnostic tool seems to be more reliable. Moreover, such a differentiated approach could help to better understand the complex pathophysiological processes of AKI and other obviously related complications. However, potential limitations exist for such multidimensional proteomic tests as used in this study. These can arise from the high inter-individual variability of the urinary proteome and its dynamic nature under the influence of nonrenal factors such as biological variance. Moreover, correcting for urinary dilution or removal of peptides with specific properties, like those with affinity to albumin, during sample preparation might introduce to some degree further analytical variability. However, in this study measures were undertaken to keep these sources of variability to a minimum. This includes:

1. Preparation of the urine sample under chaotropic and denaturating conditions that are strong enough to disrupt any protein-to-protein interaction before the removal of high molecular compounds above 20 kDa [31].
2. Use of the flow-based sieving matrix-free capillary electrophoresis for peptide separation and operation of the CE-MS under highly acidic conditions (pH 2) preventing adsorption of the peptides to the capillary surface [31].
3. Normalization of the peptide’s amplitude signals to 29 highly abundant internal collagen peptides with low variance in their amplitude signals and which are unaffected by any known disease state to compensate for differences in the dilution of individual urine samples [22].

Moreover, the peptide marker panel itself is well balanced in its composition in respect to the inclusion of peptide markers with increased or decreased urinary levels during AKI progression making classification independent from the overall peptide content of the urine [14].

A limitation of CE-MS is its highly skilled operating level and relative high operating costs making it impractical at first sight for close and cost-effective monitoring of acute events in daily clinical practice. A transfer of the peptide markers to the higher throughput, less expensive, and less skilled matrix-assisted laser desorption ionization (MALDI)-MS technology is one way to solve this problem [32]. However, since we could establish a proteomic pattern of AKI a few days prior to its clinical manifestation, a strategy where a urine sample will be drawn routinely from the catheter bag a few hours after cardiac surgery for immediate analysis by CE-MS directly at the hospital site might also be a practical strategy, as already is in place for other sophisticated diagnostic systems like flow cytometry-based diagnostics, in order to improve clinical management of post-cardiac surgery patients at least in university hospitals and large cardiac surgery reference centers.

Although this study confirms that our previously published proteomic marker model detects AKI accurately, the number of included patients is still relatively small. The wide use of CE-MS in clinical practice is not yet possible due to the aforementioned methodical and technical aspects. Since the overall method from sample preparation over CE-MS analysis to proteomic data processing is rather time consuming (>24 hours) and of high cost (approximately 300 to 800 euros per sample), we could not test all 865 patients of the initial prospective study population. Based on a sample size calculation with respect to the statistical power, we have thus included 60 patients with AKI and an equal number of patients without AKI as a control group in this validation study. Moreover, we initially did not intend to compare any parameter other than AKI between these 120 patients. Thus, for the obvious coincidence of AKI with NOMI and or death described above, we can give no further explanation or examine this with respect to our data.

## Conclusions

The recent study provided evidence that the 20-peptide marker panel originally established for AKI prediction in ICU patients also allows accurate prediction of AKI in the clinical setting of cardiac surgery. In this instance, it is superior to the prediction based on urinary NGAL and KIM-1 levels. In addition, the predictive performance for the AKI-associated endpoints, NOMI, and death reached the same high level of accuracy as for AKI. Although discrimination of higher AKIN classes was not possible in all patients, our test allows prediction of severe outcome (AKIN classes 2 and 3) in AKI. Thus, our proteomic marker model for AKI prediction may positively impact the management of ICU patients helping to improve outcome in AKI.

## Key messages

In patients undergoing elective cardiac surgery:

- Urine proteomic analysis allows accurate and early detection of AKI, even before AKI can be diagnosed by increase of creatinine as described in the current definition for AKI.
- The predictive capacity for postoperative AKI of our 20-peptide marker pattern is superior compared to the diagnosis based on urinary levels of NGAL and KIM-1. The same high level of predictive performance of the proteomic test as for AKI was reached for the AKI-associated endpoints, NOMI, and death.
- Since AKI is a common endpoint of different pathological pathways, a multifactorial diagnostic tool such as the 20-peptide marker pattern seems to be more reliable for diagnosis of AKI than diagnostic tests based on single AKI markers.

## Abbreviations

AKI, acute kidney injury; AUC, area under the ROC curve; CE-MS, capillary electrophoresis-mass spectrometry; CI, confidence interval; ICU, intensive care unit; KIM-1, kidney injury molecule-1; NGAL, neutrophil gelatinase-associated lipocalin; NOMI, nonocclusive mesenteric ischemia; ROC, receiver operating characteristic

## Additional file

Additional file 1: Table S1. Sequence information for the 20 urinary peptides included in the AKI peptide marker pattern. (PDF 10804 kb)
