# Supplementary material for: Acute kidney injury prediction in cardiac surgery patients by a urinary peptide pattern: a case-control validation study
Source: Crit Care. 2016 May 26;20:157. doi: 10.1186/s13054-016-1344-z (PMC4882859; doi:10.1186/s13054-016-1344-z)
Supplement: Additional file 1: Table S1. — Sequence information for the 20 urinary peptides included in the AKI peptide marker pattern. (PDF 10804 kb) [file 13054_2016_1344_MOESM1_ESM.pdf]

**Characteristics of the 20 peptides included in the urine peptide marker panel for AKI detection.** The table is based on peptide data published by Metzger et al. (14).

| CE-MS peptide Id[a] | CE-MS characteristics | | MS/MS amino acid sequence information | | | | | Direction of regulation in AKI |
|---|---|---|---|---|---|---|---|---|
| | Mass [Da] | CE migration time [min] | Sequence[b] | Protein name | SwissProt/ TrEMBLE name | AA[c] | Sequence-derived mass [Da] | |
| 38879 | 1439.66 | 29.82 | TIDEKGTEAAGAMF | α-1-antitrypsin | A1AT_HUMAN | 363 - 376 | 1439.66 | Up |
| 5913 | 912.52 | 20.06 | KVVNPTQK | | | 411 - 418 | 912.54 | Up |
| 16832 | 1081.64 | 20.73 | IQRTPKIQV | β-2-microglobulin | B2MG_HUMAN | 21 - 29 | 1081.66 | Up |
| 30733 | 1300.58 | 28.54 | VSGFHPSDIEVD | | | 47 - 58 | 1300.59 | Up |
| 106195 | 2716.37 | 20.19 | LLKNGERIEKVEHSDLSFSKDWS | | | 59 - 81 | 2716.38 | Up |
| 100537 | 2603.28 | 20.07 | LKNGERIEKVEHSDLSFSKDWS | | | 60 - 81 | 2603.30 | Up |
| 53216 | 1654.78 | 23.13 | SpGEAGRpGEAGLpGAKG | Collagen α-1(I) chain | CO1A1_HUMAN | 522 - 539 | 1654.79 | Down |
| 57531 | 1737.78 | 31.00 | TGSpGSpGPDGKTGPPGpAG | | | 541 - 560 | 1737.78 | Down |
| 27517 | 1250.56 | 27.93 | ApGDRGEpGPpGP | | | 798 - 810 | 1250.55 | Down |
| 130747 | 3359.58 | 31.90 | PpGADGQPGAKGEpGDAGAKGDAGPpGPAGPAGPpGPIG | | | 816 - 854 | 3359.57 | Down |
| 115491 | 2942.30 | 22.23 | ESGREGApGAEGSpGRDGSpGAKGDRGETGP | | | 1011 - 1041 | 2942.30 | Down |
| 70413 | 2007.95 | 22.10 | DGESGRpGRpGERGLpGPpG | Collagen α-1(III) chain | CO3A1_HUMAN | 230 - 249 | 2007.94 | Down |
| 61573 | 1825.79 | 20.13 | DEAGSEADHEGTHSTKR | Fibrinogen α chain | FIBA_HUMAN | 605 - 621 | 1825.78 | Down |
| 64256 | 1882.80 | 20.24 | DEAGSEADHEGTHSTKRG | | | 605 - 622 | 1882.80 | Down |
| 98089 | 2559.18 | 19.41 | DEAGSEADHEGTHSTKRGHAKSRP | | | 605 - 628 | 2559.18 | Up |
| 74187 | 2080.94 | 20.20 | DAHKSEVAHRFKDLGEEN | Serum albumin | ALBU_HUMAN | 25 - 42 | 2080.99 | Up |
| 89325 | 2356.15 | 19.52 | DAHKSEVAHRFKDLGEENFK | | | 25 - 44 | 2356.16 | Up |
| 92698 | 2427.18 | 19.58 | DAHKSEVAHRFKDLGEENFKA | | | 25 - 45 | 2427.19 | Up |
| 97301 | 2540.26 | 19.68 | DAHKSEVAHRFKDLGEENFKAL | | | 25 - 46 | 2540.28 | Up |
| 102392 | 2639.32 | 19.78 | DAHKSEVAHRFKDLGEENFKALV | | | 25 - 47 | 2639.35 | Up |

Abbreviations: AA, amino acid; AKI, acute kidney injury; CE, capillary electrophoresis; Da, Dalton; min, minutes; MS, mass spectrometry.

[a] Peptide identification number; [b] Lower case p indicates hydroxyproline; [c] Amino acid positions in the proteins primary sequence according to UniProt Knowledge Base numbering.
